# Minimally invasive pancreatoduodenectomy is associated with lower morbidity compared to open pancreatoduodenectomy: An updated meta-analysis of randomized controlled trials and high-quality nonrandomized studies

**DOI:** 10.1097/MD.0000000000016730

**Published:** 2019-08-09

**Authors:** Jia-fei Yan, Yu Pan, Ke Chen, He-pan Zhu, Qi-long Chen

**Affiliations:** Department of General Surgery, Sir Run Run Shaw Hospital, School of Medicine, Zhejiang University, Hangzhou, Zhejiang Province, China.

**Keywords:** high quality, laparoscopy, meta-analysis, minimally invasive, morbidity, pancreaticoduodenectomy, robot

## Abstract

**Background:**

Minimally invasive pancreatoduodenectomy (MIPD) is being increasingly performed as an alternative to open pancreatoduodenectomy (OPD) in selected patients. Our study aimed to present a meta-analysis of the high-quality studies conducted that compared MIPD to OPD performed for pancreatic head and periampullary diseases.

**Methods:**

A systematic review of the available literature was performed to identify those studies conducted that compared MIPD to OPD. Here, all randomized controlled trials identified were included, while the selection of high-quality, nonrandomized comparative studies were based on a validated tool (i.e., Methodological Index for Nonrandomized Studies). Intraoperative outcomes, postoperative recovery, oncologic clearance, and postoperative complications were also evaluated.

**Results:**

Sixteen studies matched the selection criteria, including a total of 3168 patients (32.1% MIPD, 67.9% OPD). The pooled data showed that MIPD was associated with a longer operative time (weighted mean difference [WMD] = 80.89 minutes, 95% confidence interval [CI]: 39.74–122.05, *P* < .01), less blood loss (WMD = −227.62 mL, 95% CI: −305.48 to −149.75, *P* < .01), shorter hospital stay (WMD = −4.68 days, 95% CI: −5.52 to −3.84, *P* < .01), and an increase in retrieved lymph nodes (WMD = 1.85, 95% CI: 1.33–2.37, *P* < .01). Furthermore, the overall morbidity was significantly lower in the MIPD group (OR = 0.67, 95% CI: 0.54–0.82, *P* < .01), as were total postoperative pancreatic fistula (POPF) (OR = 0.79, 95% CI: 0.63–0.99, *P* = .04), delayed gastric emptying (DGE) (OR = 0.71, 95% CI: 0.52–0.96, *P* = .02), and wound infection (OR = 0.56, 95% CI: 0.39–0.79, *P* < .01). However, there were no statistically significant differences observed in major complications, clinically significant POPFs, reoperation rate, and mortality.

**Conclusion:**

Our study suggests that MIPD is a safe alternative to OPD, as it is associated with less blood loss and better postoperative recovery in terms of the overall postoperative complications as well as POPF, DGE, and wound infection. Methodologic high-quality comparative studies are required for further evaluation.

## Abstract 2

Supplemental Digital Content is available in the text

## 1 Introduction

Pancreatoduodenectomy (PD) is one of the most complex procedures performed in the field of abdominal surgery, though the number of these procedures being performed has been on the rise in recent years due to the improved diagnostic capabilities, expanding indications, and formal development of pancreatic surgery training. However, despite advances in patient selection, surgical techniques, and postoperative care, morbidity still occurs in up to 40% of patients undergoing open pancreatoduodenectomy (OPD).^[1]^

Contrastingly, minimally invasive surgery (MIS) has been the main direction established in terms of surgical development in the 21st century.^[2]^ Researchers have demonstrated how MIS could be used to achieve catabatic pain, reduced morbidity, shorter hospital stays, and a rapid return to baseline performance status, with oncologic equivalent outcomes when compared to the traditional open surgery procedures,^[3–5]^ and therefore, the selection of MIS has become the professional choice of pursuit for surgeons, as well as the preferred treatment option for patients.^[6]^ With respect to pancreatic surgery, however, the development of minimally invasive pancreatoduodenectomy (MIPD) lags considerably behind that of minimally invasive distal pancreatectomy, as the latter represents a less-demanding technique without any reconstruction, whereas the former is technically demanding and should be performed only in referral centers by experienced hands. Over the past few years, the advancements in relevant devices and gained experience have gradually expanded the indications of MIPD, resulting in several centers reporting promising outcomes after MIPD was performed,^[7]^ yet the controversies and concerns regarding MIPD vs OPD still remain, which has led some researchers to address these issues objectively in the form of meta-analyses.^[8–12]^ However, in these studies, conflicting results have been found between published meta-analyses with respect to morbidity, mortality, retrieved lymph nodes (RLNs), and surgical margins. Additionally, as randomized controlled trials (RCTs) published on this topic are scarce, these meta-analyses further include all nonrandomized controlled trials (NRCTs) available to pool the outcomes together. On the one hand, however, poor-quality NRCTs may have exaggerated the effect magnitude of an intervention, either by their intrinsic flaws or external factors such as publication bias, and therefore, meta-analyses based on such studies have not been adequate to examine the advantages and disadvantages of this emerging technique. On the other hand, there has also been evidence that the estimates derived from high-quality NRCTs may be similar to those derived from RCTs.^[13]^ Therefore, we designed an updated study by pooling the data from all of the available RCTs and high-quality NRCTs published to date, to evaluate the safety and efficacy of MIPD as an acceptable alternative to OPD.

## 2 Materials and methods

Our current meta-analysis was undertaken in accordance with the Cochrane Handbook for Systematic Reviews of Interventions and the Preferred Reporting Items for Systematic Reviews and Meta-Analyses (PRISMA) guidelines.^[14]^ This study did not require ethical approval as it was a review of the existing published literature and did not involve the handling of individual patient data.

### 2.1 Literature search

A PubMed, Embase, and Google Scholar database search were each performed to identify all published comparative studies available that analyzed and compared MIPD to OPD. Keywords included the terms “minimally invasive,” “laparoscopic,” “robotic,” “Da Vinci,” “pancreaticoduodenectomy,” “Whipple,” “PD,” and “pancreatic resection,” with the search restricted to human studies published only between January 1994 and January 2019. Furthermore, MIPD here included both laparoscopic pancreatoduodenectomy (LPD) and robot-assisted pancreatoduodenectomy (RPD) data. References from relevant articles and reviews were manually searched for, while the language of publication was confined to English.

### 2.2 Quality assessment

Checklists were used by reviewers for data extraction and assessment of the methodologic quality, with the methodologic quality of the eligible RCTs assessed by the Jadad scale, which included all RCTs in the analysis, and that of the NRCTs assessed by the Methodological Index for Nonrandomized Studies (MINORS),^[15]^ a tool developed by a group of practicing surgeons in France and validated specifically for such NRCT evaluations. Certain modifications were introduced to the MINORS to meet the needs of our study, which have been listed in Supplementary Table 1. In total, 8 items were evaluated, each with a maximum score of 16 points, the studies with 12 or more points were considered to be high quality and were included in our meta-analysis, while those with <12 points were considered poor quality and were excluded.

### 2.3 Data extraction

Two investigators independently tabulated the extracted data and a double-check procedure was also performed to ensure its accuracy, following which a manager subsequently inputted the data into a spreadsheet. Duplications in the data were identified by matching both the authors’ names and publication center, and any overlaps between authors or centers were resolved by selecting the higher quality or more recent literature published by them.

### 2.4 Variables and endpoints

Basic demographics: 1st author, publication year, and total number of patients in both groups.

Intraoperative parameters: operative time, estimated blood loss, and blood transfusion rate.

Postoperative parameters: length of hospital stay, morbidity, postoperative pancreatic fistula (POPF), delayed gastric emptying (DGE), postpancreatectomy hemorrhage (PPH), wound infection, reoperation rate, and mortality.

Oncologic clearance: RLNs and surgical margins.

The intention-to-treat (ITT) analysis was investigated, with POPF, DGE, and PPH diagnosed in accordance with the International Study Group for Pancreatic Fistula (ISGPF) criteria.^[16–18]^ In our study, a clinically significant POPF was defined as ISGPF grade B/C.^[16]^ Moreover, the Clavien-Dindo classification for postoperative morbidity was also checked,^[19]^ in which the major complications were established as grades III to V.

### 2.5 Statistical analysis

Statistical analyses were performed using odds ratios (ORs) with 95% confidence intervals (CIs) for dichotomous variables and weighted mean differences (WMDs) with 95% CIs for continuous variables. The statistical mean and standard deviations, medians and ranges, or interquartile ranges were not estimated as described by Hozo et al,^[20]^ as this method may have led to a deviation from the true value, especially if the sample size was small or the samples exhibited significant skewness. Moreover, the heterogeneity among studies was assessed using a Chi-squared test-based *Q*-statistic and was considered statistically significant for *P* < .10, while the effect of heterogeneity was quantified using *I*^2^ = 100% × (*Q* – df)/*Q*, with the *I*^2^ ranges between 0% and 100% and the *I*^2^ values of 25%, 50%, and 75% defined as low, moderate, and high estimates, respectively. If data were not significantly heterogeneous (*P* > .05 or *I*^2^ < 50%), the pooled effects were calculated using a fixed model, in contrast to the significantly heterogeneous data, for which a random model was used. Furthermore, a subgroup analysis that categorized MIPD into LPD and RPD was also conducted to determine the accurate advantages and consistency of both the procedures. Lastly, any potential publication bias was determined by conducting an informal visual inspection of the funnel plots. The RevMan 5.3 software (Copenhagen: The Nordic Cochrane Centre, The Cochrane Collaboration, 2014) was implemented for the statistical analyses in our study, with a 2-tailed value of *P* < .05 being considered significant.

## 3 Results

### 3.1 Studies selected and quality assessment

**Figure 1:**
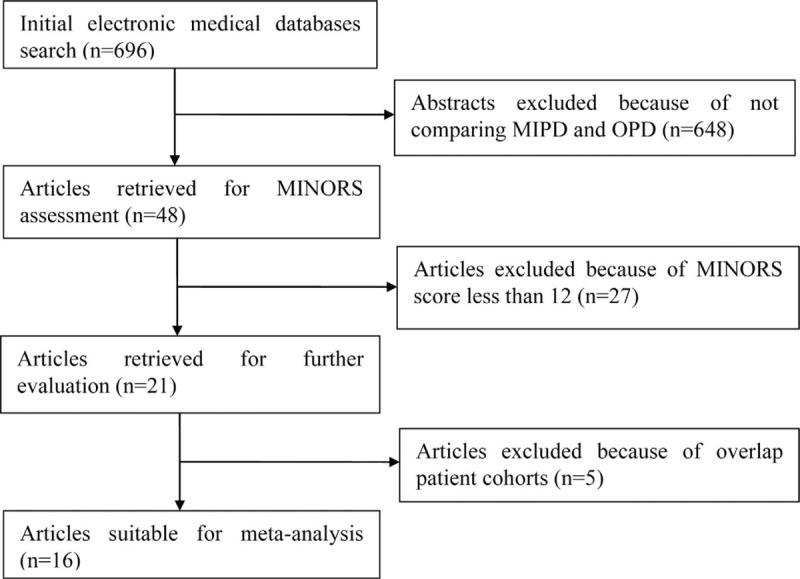
Flow chart of literature search strategies.

Data extraction from the various electronic databases yielded an initial total of 696 abstracts. After the exclusion of nonrelevant citations, 48 potentially relevant citations remained for full-text screening, which included 1 RCT and 47 NRCTs, all published between 2009 and 2018. Twenty-seven of these NRCTs were then excluded after our quality assessment was performed, due to MINORS scores of <12,^[21–47]^ followed by 5 more NRCTs, which contained data that overlapped with data from other included studies.^[48–52]^ Thus, 1 RCT^[53]^ and 15 NRCTs were finally included in the present study.^[54–68]^ A flowchart of the aforementioned literature search strategies is shown in Figure 1. The single RCT received a Jadad score of 3.^[53]^ The assessment of the NRCTs is summarized in Table 1. Generally, the poor-quality NRCTs suffered from methodologic drawbacks frequently seen in retrospective designs, incomplete outcomes reports, and perioperative clinical mismanagement. The major features of the studies included in the meta-analysis are summarized in Table 2. A total of 3168 patients were included in the analysis, of which 1018 (32.1%) underwent MIPD and the remaining 2150 (67.9%) underwent OPD. With respect to the MIPD approach taken, 10 studies were reported as performing LPD,^[53,54,57,58,60,61,63,65,67,68]^ whereas another 6 performed RPD.^[55,56,59,62,64,66]^

**Table 1:**
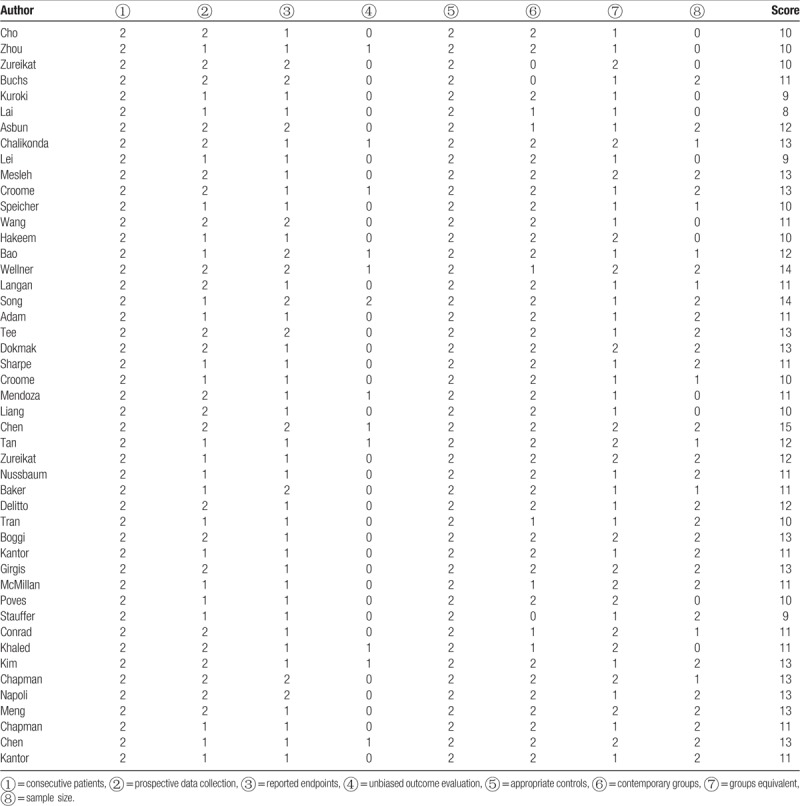
Modified Methodological Index for Nonrandomized Study score of initial eligible nonrandomized comparative studies.

**Table 2:**
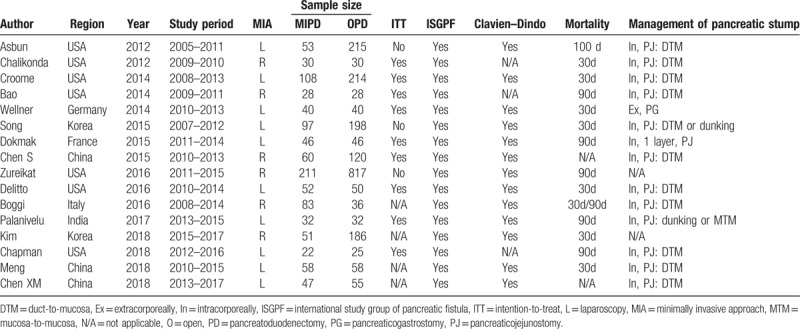
Summary of studies included in the meta-analysis.

### 3.2 Meta-analysis

All eligible parameters were pooled for the meta-analysis. The results of this are listed in Table 3.

**Table 3:**
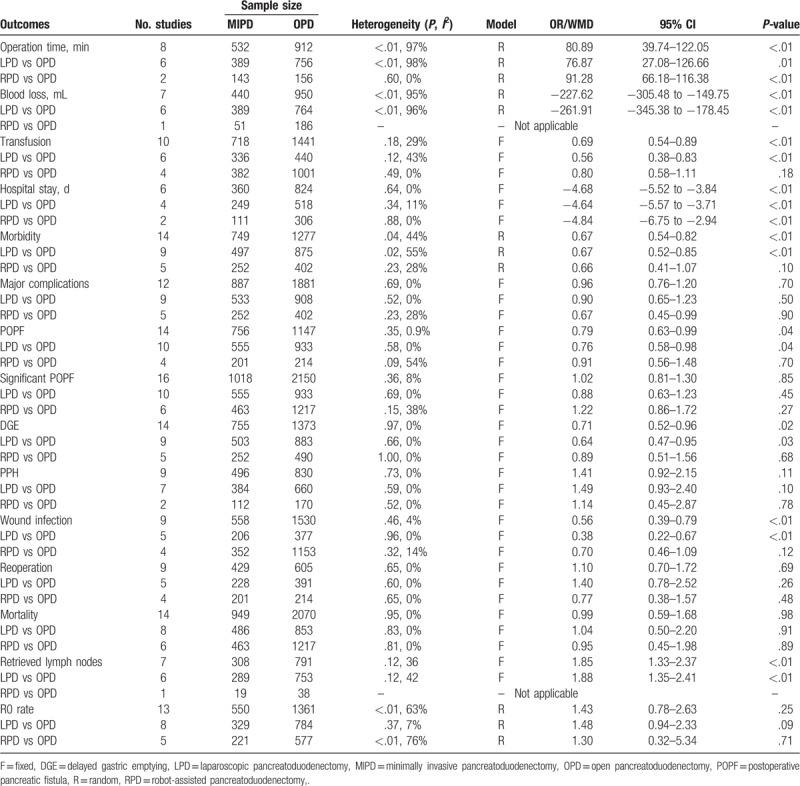
Results of the meta-analysis.

#### 3.2.1 Intraoperative effects

The pooled data showed that MIPD was associated with a longer operative time (WMD = 80.89 minutes, 95% CI: 39.74–122.05, *P* < .01), less blood loss (WMD = −227.62 mL, 95% CI: −305.48 to −149.75, *P* < .01), and lower transfusion rates (OR = 0.69, 95% CI: 0.54–0.89, *P* < .01). Additionally, statistically significant results with respect to between-study heterogeneity were identified in terms of the operative time (*I*^2^ = 97%, *P* < .01) and blood loss (*I*^2^ = 95%, *P* < .01), but not the transfusion rate (*I*^2^ = 29%, *P* = .18).

#### 3.2.2 Postoperative clinical course

**Figure 2:**
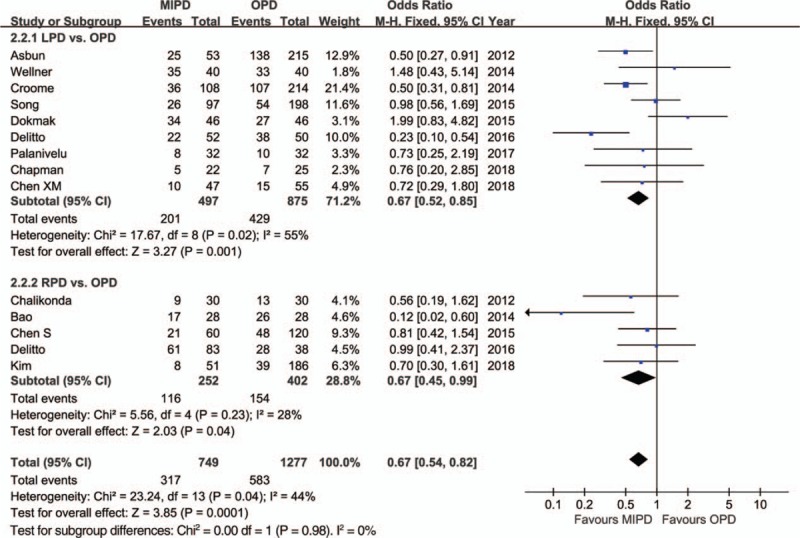
Forest plot of the meta-analysis: morbidity.

**Figure 3:**
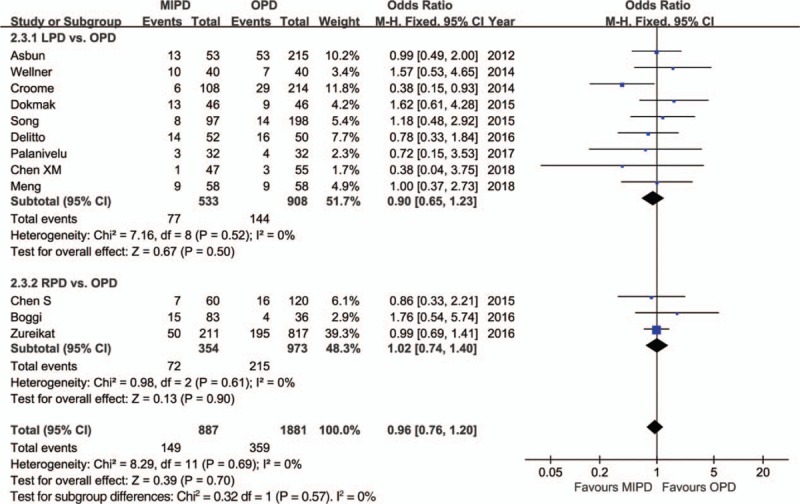
Forest plot of the meta-analysis: major complications. CI = confidence interval, LPD = laparoscopic pancreatoduodenectomy, MIPD = minimally invasive pancreatoduodenectomy, OPD = open pancreatoduodenectomy, RPD = robot-assisted pancreatoduodenectomy.

**Figure 4:**
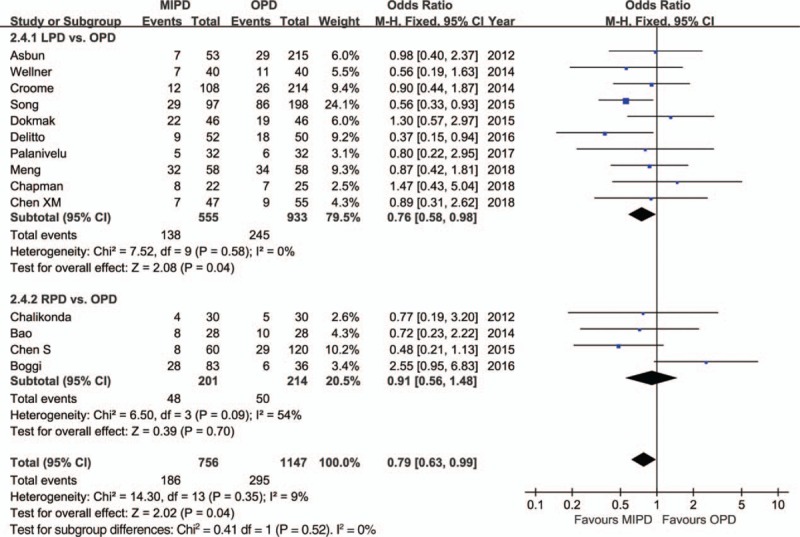
Forest plot of the meta-analysis: overall postoperative pancreatic fistula. CI = confidence interval, LPD = laparoscopic pancreatoduodenectomy, MIPD = minimally invasive pancreatoduodenectomy, OPD = open pancreatoduodenectomy, RPD = robot-assisted pancreatoduodenectomy.

**Figure 5:**
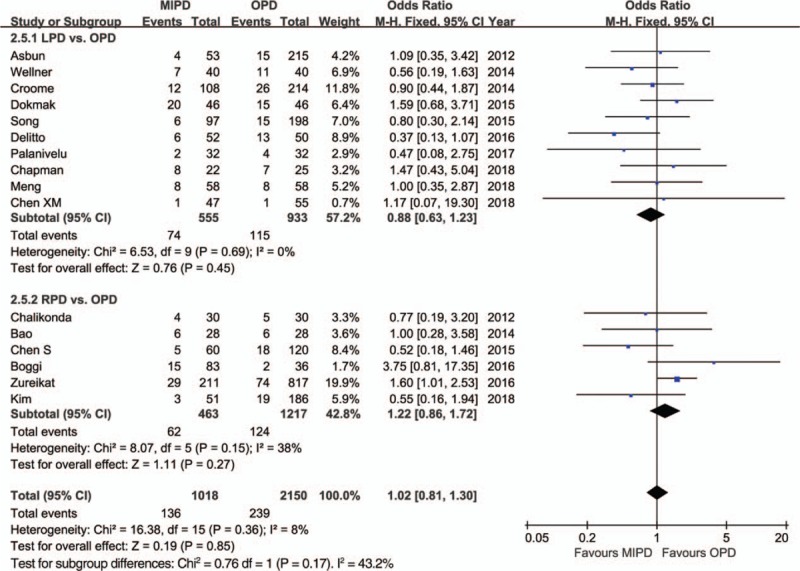
Forest plot of the meta-analysis: clinically significant postoperative pancreatic fistula. CI = confidence interval, LPD = laparoscopic pancreatoduodenectomy, MIPD = minimally invasive pancreatoduodenectomy, OPD = open pancreatoduodenectomy, RPD = robot-assisted pancreatoduodenectomy.

**Figure 6:**
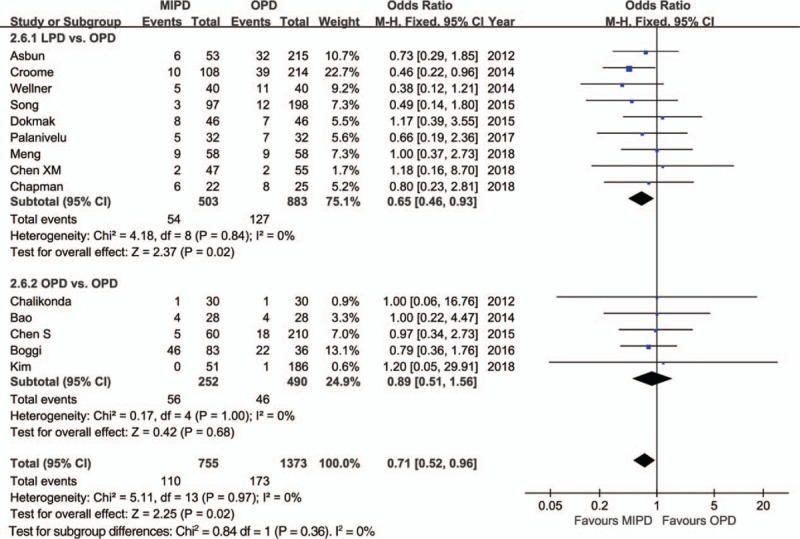
Forest plot of the meta-analysis: delayed gastric emptying. CI = confidence interval, LPD = laparoscopic pancreatoduodenectomy, MIPD = minimally invasive pancreatoduodenectomy, OPD = open pancreatoduodenectomy, RPD = robot-assisted pancreatoduodenectomy.

**Figure 7:**
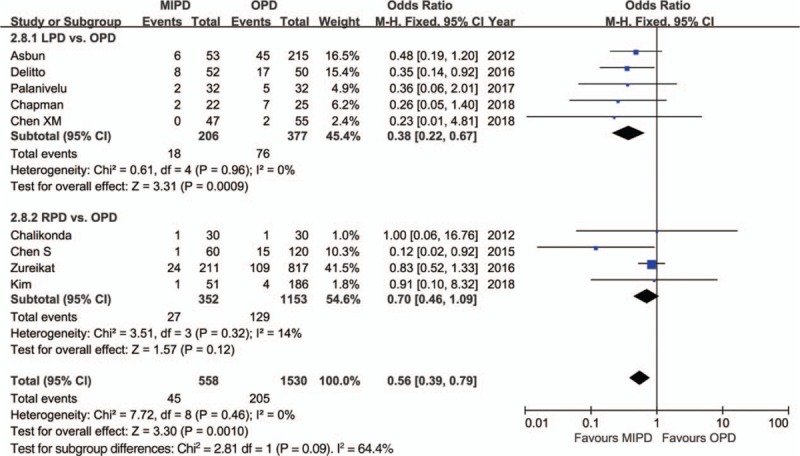
Forest plot of the meta-analysis: wound infection. CI = confidence interval, LPD = laparoscopic pancreatoduodenectomy, MIPD = minimally invasive pancreatoduodenectomy, OPD = open pancreatoduodenectomy, RPD = robot-assisted pancreatoduodenectomy.

The pooled data further showed a shorter length of hospital stay with respect to MIPD (WMD = −4.68 days, 95% CI: −5.52 to −3.84, *P* < .01) without significant heterogeneity (*I*^2^ = 0%, *P* = .64), and furthermore, the pooled analysis indicated that the rate of overall morbidity was significantly lower in the MIPD group (OR = 0.67, 95% CI: 0.54–0.82, *P* < .01) with moderate heterogeneity (*I*^2^ = 44%, *P* = .04) (Fig. 2). Contrarily, our analysis revealed that there was no significant difference in terms of major complications between the MIPD and OPD groups (OR = 0.96, 95% CI: 0.76–1.20, *P* = .70; heterogeneity: *I*^2^ = 0%, *P* = .69) (Fig. 3). The pooled data also showed reduced POPF rates in the MIPD group (OR = 0.79, 95% CI: 0.63–0.99, *P* = .04) without significant heterogeneity (*I*^2^ = 9%, *P* = .35), which was mainly due to the contributions associated with LPD rather than those of RPD (Fig. 4); in contrast, there was no significant difference between the incidences of clinically significant POPFs (OR = 1.02, 95% CI: 0.81–1.30, *P* = .85; heterogeneity: *I*^2^ = 8%, *P* = .36) (Fig. 5). The pooled data additionally showed a significant difference with respect to DGE that favored MIPD, which was also mainly due to LPD (OR = 0.71, 95% CI: 0.52–0.96, *P* = .02; heterogeneity: *I*^2^ = 0%, *P* = .97) (Fig. 6), and also indicated that those patients who underwent MIPD suffered less in terms of wound infection (OR = 0.56, 95% CI: 0.39–0.79, *P* < .01) without significant heterogeneity (*I*^2^ = 0%, *P* = .46) (Fig. 7). Lastly, our meta-analysis showed there was no statistically significant difference in the incidences of PPH, reoperation rate, and mortality between the 2 groups (Table 3).

#### 3.2.3 Oncologic clearance

The pooled data with respect to the RLNs showed that there was an increase in RLN associated with MIPD compared to OPD (WMD = 1.85, 95% CI: 1.33–2.37, *P* < .01). Moreover, the pooled data indicated a comparable R0 rate between the groups (OR = 1.05, 95% CI: 0.79–1.40, *P* = 0.74). There was no definitive statistically significant result with respect to between-study heterogeneity in RLN (*I*^2^ = 42%, *P* = .12); however, statistically significant between-study heterogeneity was identified in the R0 rate (*I*^2^ = 63%, *P* < .12).

### 3.3 Publication bias

**Figure 8:**
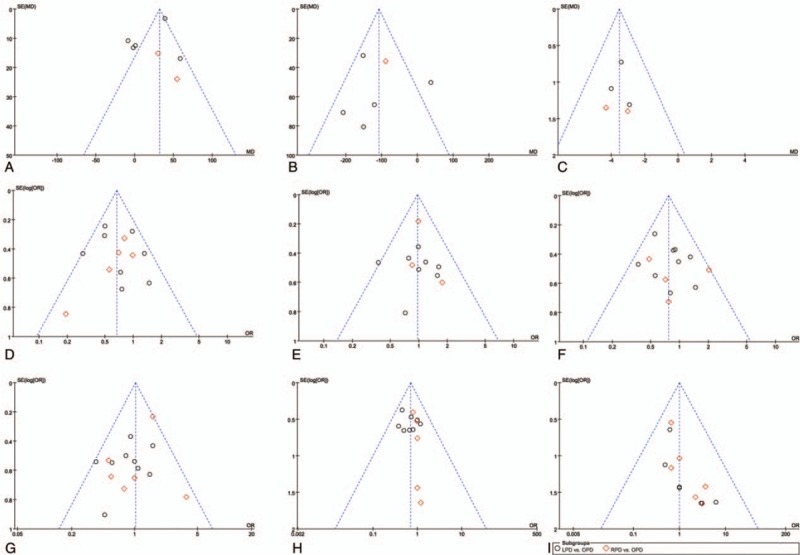
Funnel plots of the meta-analysis: (A) operative time, (B) blood loss, (C) hospital stay, (D) morbidity, (E) major complications, (F) postoperative pancreatic fistula (POPF), (G) significant POPF, (H) delayed gastric emptying, (I) mortality.

Funnel plots were drawn for each outcome and subsequently assessed for symmetry. The funnel plots of the publications in this study were found to be symmetrical, which suggested limited or no publication bias (Fig. 8).

## 4 Discussion

Our meta-analysis revealed that significant reductions in intraoperative blood loss, frequency rate of transfusion, length of hospital stay, and the incidences of overall POPF, DGE, and wound infection were observed after MIPD; the postoperative mortality, major complications, significant POPF, incidences of PPH, reoperation rate, and R0 rate were comparable to the OPD group data; and a prolonged operation time as well as an increase in RLNs were observed in the patients with MIPD. These findings are not in line with those of several previously conducted meta-analyses^[8–12]^; however, the present study was conducted including the RCT and only high-quality NRCTs, as well as a comprehensive investigation of the short-term outcomes. We believe the results from our meta-analysis highlight the safety and efficacy of MIPD more robustly than any other publication has to date.

The most important concern regarding the development of any new surgical approach is that of the patient's safety. While previous meta-analyses on the overall postoperative morbidity after MIPD have shown conflicting results, in the present analysis, the overall complication rate was lower for MIPD than OPD, despite the moderate heterogeneity among the included studies (*I*^2^ = 44%). However, the pooled data here indicated no difference in major morbidity, and similarly, our meta-analysis revealed that the overall POPFs after MIPD were fewer, whereas clinically significant POPFs were comparable to that of OPD. Thus, MIPD may be beneficial in reducing minor complications and grade A POPFs when compared to OPD. POPFs are widely regarded as the most common and ominous complications to occur following pancreatic resection, and a significant POPF is the greatest contributor to the major morbidity and mortality that occurs following PD.^[69]^ A high-quality pancreatic anastomosis would firmly protect patients from the occurrence of severe POPFs, and as such, various surgical procedures have been devised to aid in their prevention. Although there have been debates on which procedure (e.g., pancreaticojejunostomy or pancreaticogastrostomy, duct-to-mucosa or invagination anastomosis, and so on) is optimal, they can all be meticulously performed by minimally invasive methods.^[7]^ Since the true risk factors of significant POPFs (such as soft pancreatic parenchyma, high-risk disease pathology, and small pancreatic duct size) have been recognized,^[70]^ the comparably severe POPFs rates can be explained, as the high-quality studies included in this meta-analysis have a convincing baseline characteristic comparability. Grade A fistulas are transient and asymptomatic, and have elevated drain amylase levels. The possible reasons for lowering a fistula from grade A in MIPD can be explained as follows: intraoperative high-resolution images help to meticulously separate and protect the pancreatic parenchyma; MIPD has less of an influence on the peripheral organs and peritoneum, leading to the reduced occurrence of seroperitoneum; and the effect of drainage is better in the minimally invasive groups.

The DGEs are the 2nd most common postoperative complications to occur after PD. Although not a life-threatening complication, it delays oral intake, prolongs the hospital stay, diminishes nutritional status, decreases the quality of life, and increases the total costs of hospitalization.^[71]^ Our pooled data revealed that DGE occurred at a lesser rate in the MIPD than in the OPD group without statistical heterogeneity. The potential reasons for this advantage of MIPD are mainly attributed to the use of a high-solution laparoscope and the meticulous attention to technique, which involves: alleviation of gastric dysrhythmias due to fewer minor POPFs and ascites; ameliorative pyloric or antral ischemia due to the reservation of small vessels; and mitigant pylorospasm secondary to the denervation of the stomach and duodenum or jejunum.

Nevertheless, a reduction in minor POPFs and DGEs does not tell the complete story of fewer overall complications after MIPD. As one of the most complex procedures in abdominal surgery, PD involves multiple organ systems and is likely to cause more medical complications than other similar operations. It is well known that major abdominal surgery has a detrimental effect on respiratory function, particularly with upper abdominal surgery. MIS, however, reduces the risk of such pulmonary complications with its associated mild postoperative pain and the opportunity for earlier ambulation.^[72]^ Additionally, a lower wound infection rate of MIPD was observed in our meta-analysis, possibly due to the shorter wound length associated with MIPD, and consequently, the incidence rate of postoperative hernia occurring may be less common than that of OPD. Since postoperative hernias usually occur after a long period postsurgery, and the follow-up time in many of the original studies may not have been adequately long, some researchers might not have observed this complication during the course of their research, and thus, only 1 study was found to have documented an incisional hernia.^[56]^ These findings of comparable major complications and significant POPFs were in accordance with the similar mortality and reoperation data observed in MIPD compared to OPD. The low reported mortality rate could be considered to be an indicator of the safety of this technique.

In line with previous meta-analyses, a longer operative time and less blood loss were observed in MIPD. Although none of the MIPD studies identified any adverse outcomes, a recent study from the American College of Surgeons NSQIP demonstrated that longer operative times were independently associated with worse perioperative outcomes after pancreatic resection.^[73]^ Since a longer duration of surgery might also indicate intraoperative difficulties or surgical inexperience, those surgeons who wish to embark on an MIPD program should have a clear plan, including the recruitment of trained providers and/or training of the local team as well as avoiding complex cases (such as obese patients, neoadjuvant treatment, preoperative biliary stenting, vascular involvement, and/or concurrent organ resections) during the learning curve.^[7,73]^

The time required for postoperative recovery has been reported in only a few of the studies, precluding the meta-analysis. Two studies here reported significantly faster returns to ambulation and bowel recovery following MIPD compared to OPD.^[59,67]^ In line with previous meta-analyses, a shorter hospital stay was also observed for patients with MIPD, with fewer complications, reduced pain and use of analgesic drugs,^[49,61,67]^ sound nutritional status,^[59]^ and earlier activities all contributing to shorten the hospital stay.

The role of MIPD in the setting of malignancy is currently under evaluation, and thus, good quality reproducible data on this is limited. Several studies have reported disease free and/or overall survival with comparable or favorable outcomes with MIPD as compared to OPD^[57,59,61,63,65,67]^; however, the different malignancies among various patients, short-term assessment, and lack of other data to precisely validate these findings prompts the need for further studies. The majority of published studies instead focus on the surrogates of an oncologic resection, namely lymph node retrieval and surgical margin status. Our analysis revealed that MIPD was associated with an increase in RLNs and comparable R0 resection rates. The removal of a sufficient number of lymph nodes could enhance the accuracy of staging and regional disease control. The advantages of high-resolution images, multidimensional vision, and meticulous manipulation could help MIPD facilitate lymphadenectomy. However, the increase in lymph node retrieval associated with the MIPD group should be interpreted with caution; consideration of a selection bias is important, as easy-to-surgery patients are being chosen for the novel technique. Furthermore, different pathologic processing techniques of the surgical specimen have also been shown to yield significantly different lymph node counts.^[74]^ Another potential advantage of MIPD here may be the earlier receipt of adjuvant chemotherapy thanks to the shorter hospital stay, but definitive evidence would still be needed for this. Based on a similar theory, major complications could negatively impact the survival of patients undergoing curative-intent pancreatectomy, as morbidity influences multimodality therapy completion.^[75]^ We cannot conclusively confirm whether MIPD has such aforementioned advantages, since major complications were also similarly identified from our pooled data. Thus, the paucity of data with respect to oncologic outcomes in MIPD underscores the need for careful and prospective scrutiny with regards to the long-term outcomes.

Our study assesses the surgical outcomes of patients undergoing PD, comparing the results of the minimally invasive approach to the open approach. The methodologic advantages of this meta-analysis include that it firstly evaluated the quality of all potential studies, and only then did it proceed to these methodologic high-quality comparative studies for further evaluation, nine of which were also case-matched studies.^[53,55,56,58–61,63,67]^ This study provides the highest possible level of evidence despite the shortage of RCTs. On the contrary, the results of this meta-analysis should also be interpreted with caution due to its several limitations, which are as follows. Firstly, the significant heterogeneity among the different studies regarding several parameters subjects our results to potential bias. There was inevitably a selection bias in the published literature, as the baseline characteristics of the patients and the indications for operative procedures in the 2 groups were not fully equal in all studies, tending to favor MIPD. Moreover, a total of 16 studies were included in our meta-analysis, with almost half of them originating from the United States (7 studies). This information implies that a publication bias possibly exists in our study. Another potential source of a publication bias is associated with the articles that were not published publicly. Secondly, clinical heterogeneity associated with our meta-analysis requires attention. The surgical techniques performed were variable in both the OPD and MIPD groups. Other factors include diverse areas, different diseases, varied severity among patients, and so on, and additionally, some endpoints have different measurements (e.g., blood loss) and not all of the articles used the Clavien-Dindo classification of surgical complications.^[55,56,65]^ Thirdly, the minimally invasive arms used, especially those of robotic cohorts, in most if not all of these institutions represent their initial surgical experiences, which could subsequently introduce another bias against the MIPD outcomes. Contrarily, it should be emphasized that most of the studies should originate from experts and pioneering centers, resulting in better outcomes for this specialization in pancreatic surgery.^[76]^ Thus, the conclusions drawn here may not be feasible to implement in smaller centers. Conclusively, methodologic high-quality RCTs using standardized reporting of outcomes are required and strongly encouraged.

## 5 Conclusion

This study suggests that MIPD is a safe alternative to OPD, as it is associated with less blood loss and better postoperative recovery in terms of a shorter hospital stay and fewer overall postoperative complications. However, this technique also has a longer operative time compared to the open procedure. Although a thorough evaluation of the short-term and long-term oncologic outcomes of MIPD was not possible here, the rate of margin positivity and number of RLNs were either comparable to, or even better than those observed in OPD. Improved levels of evidence, standardized reporting of outcomes, and ensuring the inclusion of proper RCTs in the meta-analysis are the next challenges that face this promising technique.

## Author contributions

**Conceptualization:** Jia-fei Yan.

**Data curation:** Yu Pan.

**Formal analysis:** Jia-fei Yan.

**Funding acquisition:** Qi-long Chen.

**Investigation:** Ke Chen, He-pan Zhu.

**Methodology:** Jia-fei Yan, Qi-long Chen.

**Resources:** He-pan Zhu.

**Software:** Yu Pan.

**Supervision:** Qi-long Chen.

**Writing – original draft:** Jia-fei Yan.

**Writing – review & editing:** Qi-long Chen.

## Supplementary Material

Supplemental Digital Content
