# Supplementary material for: Minimally invasive pancreatoduodenectomy is associated with lower morbidity compared to open pancreatoduodenectomy: An updated meta-analysis of randomized controlled trials and high-quality nonrandomized studies
Source: Medicine (Baltimore). 2019 Aug 9;98(32):e16730. doi: 10.1097/MD.0000000000016730 (PMC6708972; doi:10.1097/MD.0000000000016730)
Supplement: Supplemental Digital Content [file medi-98-e16730-s001.docx]

**Supplementary Table 1** Modiﬁed MINORS Scale Used for Quality Assessment of NRCT.

|  | **Points** | | |
| --- | --- | --- | --- |
|  | **0** | **1** | **2** |
| **Consecutive Patients*** | Not reported | Patients are not consecutive | Patients are consecutive |
| **Prospective Data Collection** | Not reported | Data is obtain from retrospective  review of medical history | Data is obtained from prospectively maintained database |
| **Reported Endpoint**s† | Not reported | Incomplete outcomes reported | Complete outcomes reported |
| **Unbiased outcome evaluation**‡ | No perioperative protocol for clinical management | Incomplete protocol for perioperative clinical management | Complete protocol for perioperative clinical management |
| **Appropriate controls**§ | Not reported | Incomplete report of the standard intervention | Complete report of the standard intervention |
| **Contemporary Groups** | Not reported | Study group compared with historical control group | Study group compared with contemporary control group |
| **Groups Equivalent** | Lack of comparability between groups | Partial baseline characteristics were comparable | All baseline characteristics were comparable |
| **Sample Size** | Less than 20 MIPD cases | More than 20 but less than 40 MIPD cases | More than 40 MIPD cases |

*Considered consecutive if there was clear beginning and end time of the research.

†Considered complete if interested outcomes were all reported.

‡Considered complete only if there was protocol for analgesic administration, resume of oral intake or hospital discharge.

§Considered complete if surgical methods were reported properly.
